# Is facultative sex the best of both worlds in the parasitoid wasp *Lysiphlebus fabarum?*

**DOI:** 10.1098/rsos.242162

**Published:** 2025-05-21

**Authors:** Rebecca A Boulton

**Affiliations:** ^1^ Biological and Environmental Sciences, University of Stirling, Stirling, UK; ^2^ Laboratory of Genetics, Wageningen University and Research, Wageningen, The Netherlands

**Keywords:** asexual reproduction, parthenogenesis, facultative sex, costs of sex

## Abstract

The prevalence of sexual reproduction has long puzzled evolutionary biologists. This is because asexual parthenogenesis is a more efficient mode of reproduction, and all-else-being-equal, should predominate over sex. Asexual reproduction is not without its disadvantages though, the lack of genetic recombination can render parthenogenetic lineages vulnerable to extinction under environmental change, or compromise fitness owing to the buildup of deleterious recessive mutations. Facultative sex, where individuals retain the ability to reproduce sexually and asexually, has been touted as ‘the best of both worlds’, providing the long-term genetic benefits of sex without the short-term costs. In this study, I found that parthenogenetic females of the sexually polymorphic aphid parasitoid *Lysiphlebus fabarum* readily engage in facultative sex, but facultative sex was not the ‘best of both worlds’ owing to elevated rates of reproductive failure compared to obligate sex and parthenogenesis. By contrast, obligately sexual females had increased fecundity compared to asexual females. In *L. fabarum,* it seems that all is not equal; the fecundity advantage that obligate sex provides, along with the costs of occasional facultative sex by asexuals must be factored in when attempting to understand why and how different reproductive modes coexist in nature.

## 1. Introduction

Understanding how sex became the dominant reproductive strategy in eukaryotes has long been a major theme in evolutionary biology [1–3]. The initial interest in this question arose from the observation that asexual reproduction should, in theory, predominate because it is a far more efficient way to transmit copies of one’s own genome to the next generation. An asexually reproducing individual is expected to have double the genetic representation of itself in the next generation compared to an equivalent sexual individual. This became known as the ‘twofold cost of sex’ [1]; early explanations considered ‘genome dilution’ to be the cause of this cost, whereby a sexually reproducing female ‘dilutes’ her genetic contribution to the next generation, halving her fitness compared to an asexual female. It is now accepted that ‘genome dilution’ is not usually the source of the ‘twofold’ cost of sex (as only genes for reproduction matter in this context, see [4] for an in-depth explanation), instead the twofold cost arises because sexual individuals must ‘waste’ resources on producing males, which do not efficiently convert resources to offspring [4]. When the sex ratio is 50 : 50, sexuals produce half as many daughters as asexuals, so the twofold cost of sex is sometimes known as the ‘twofold cost of males’ [1–4]. Beyond the twofold cost of males, sexual reproduction carries multiple additional costs that may affect its evolutionary maintenance. These include direct risks to survival as a result of sex (i.e. sexually transmitted diseases, physical damage owing to copulation, increased predation risk and reduced feeding) but also indirect costs that occur owing to genetic incompatibilities between mates, for instance, under both inbreeding or outbreeding [4]. Given these costs, how then did sex become more prevalent than asexual reproduction in eukaryotes after it evolved over 2 billion years ago? Also, more recently, given that asexual reproduction readily re-evolves in eukaryotes [2] why does sex still dominate? Historically, two broad models which encompass different types of benefits have been proposed to answer these questions. The first group are the mutational models, which emphasize the importance of sex in exposing mutations to selection, so that deleterious mutations can be purged, and beneficial mutations can be fixed more effectively [3,5–8]. The second group are ecological models, which emphasize the benefits of sex for adaptive potential; by creating new gene combinations sex facilitates adaptation under environmental change [9]. Mutational and ecological benefits of sex are not mutually exclusive however and can interact in complex and context-dependent ways, which may explain why phylogenetic patterns point to reproductive mode being more evolutionarily labile than expected in the eukaryotes [2–4,10,11].

The picture becomes more complex in light of mounting evidence that many species thought only to reproduce through obligate sex or asexual reproduction are actually sexually plastic [10]. Many species reproduce asexually only some of the time, either as an obligate part of their lifecycle (as in cyclical parthenogens like many aphids and *Daphnia* [11]), facultatively when a suitable mate can not be found [12], or sometimes spontaneously (so-called *Tychoparthenogenesis* [2,13]). Facultative sex has been touted as the ‘best of both worlds’ as it can, in theory, provide both the short-term demographic benefits of asexual reproduction and the long-term genetic benefits of sex [11]. Yet, facultative parthenogenesis could also expose populations to many other direct and indirect costs of sex. When sex is facultative the costs of genome dilution can come into play (as alleles which determine the reproductive mode can become combined in hybrid offspring), and outbreeding depression may counter the benefits of recombination when sex happens rarely between genetically distinct lineages. Integrating sexual plasticity in all its forms into theoretical and phylogenetic models which aim to reconstruct the evolution of reproductive mode is the obvious next step in answering the longstanding question ‘why sex?’, but this requires more data, not only about the occurrence of facultative sex (which is often cryptic) but also its costs and benefits relative to obligate strategies [4].

The sexual–asexual aphid parasitoid wasp *Lysiphlebus fabarum* offers an ideal system to explore whether facultative sex really does represent ‘the best of both worlds’. We already have a comprehensive understanding of the molecular basis and population genetics of reproductive mode in *L. fabarum* [14–16]. Asexual reproduction seems to have initially arisen 0.5 million years ago from ancestral obligate sex in *L. fabarum,* and it is determined by the inheritance of a single recessive allele which results in parthenogenesis by central-fusion automixis (the haploid products of meiosis I fuse to form diploid gametes which develop without fertilization [16,17]). While this form of parthenogenesis does maintain heterozygosity to some extent, because recombination still occurs, asexual lineages can only become more homozygous, eventually resulting in inbreeding depression and associated reductions in fitness [2]. However, population genetic studies show that parthenogenetic populations of *L. fabarum* have high levels of heterozygosity and allelic variation even compared to sexuals [14,15]. This genetic diversity has been proposed to exist because of occasional bouts of rare sex by normally asexual females, as well as ‘contagious’ parthenogenesis, where rare males produced by asexual females cross with sexual females, creating new asexual lineages [14,15]. Regardless of the mechanism, this genetic variation is thought to underlie the success of asexual reproduction in this species, resulting in parthenogenetic populations dominating across Europe [14,15]. Obligately sexual populations are less commonly encountered in the field, but sex does persist in *L. fabarum*, and occurs in sympatry with asexuality in some regions [17,18].

This study aims to test whether facultative sex occurs in *L. fabarum* and if it does, whether it represents the ‘best of both worlds’, providing the short-term benefits of asexual reproduction and long-term genetic benefits associated with sex [12]. To do this, I gave female *L. fabarum* from seven asexual lines and one outbred sexual population (all collected from regions of sympatry) aphid hosts to parasitize after (i) being exposed to males (from the sexual population), or (ii) being kept in isolation (because of haplodiploidy sexual females can produce unfertilized eggs that develop as male offspring). I found that asexual females readily copulated when they were exposed to males and so in a follow-up experiment, I genotyped their G1 offspring to test whether mating reflects a behavioural vestige of sexual reproduction without fertilization or whether these females can still reproduce sexually. I measured the reproductive success of obligately sexual, asexual and facultatively sexual females over two generations. This approach allowed me to measure the ‘many costs of sex’, rather than just the ‘twofold’, as advocated by Lehtonen *et al.* [4], providing a more comprehensive understanding of the costs and benefits of obligate and facultative sex in this system.

## 2. Methods

### 2.1. Study system

In the aphid parasitoid *L. fabarum* both sexual and asexual populations exist, separately as well as in sympatry in some regions [14,15]. Asexual reproduction is inherited via a single recessive allele which appeared approximately 0.5 million years ago [14]. Inheritance of two copies of the asexual allele results in parthenogenetic asexual reproduction through central-fusion automixis, where the products of meiosis I fuse and recombine, restoring diploidy without integrating paternal DNA [16]. A panel of microsatellite markers has been developed to study the population genetics of *L. fabarum,* one of which, *Lysi07*, shows perfect linkage to the recessive genetic factor associated with asexual reproduction [18].

### 2.2. Laboratory rearing

This study used females from seven asexual lines and males and females from an outbred obligately sexual population of the aphid parasitoid *L. fabarum*. These lines were provided by Prof Christoph Vorburger (EAWAG, Zürich, Switzerland), who has been maintaining them since they were collected between 2006 and 2017 (see [16] for details). All asexual and sexual lines but one (IL06-658) were collected from locations across Switzerland (table 1). The wasps have been cultured at the University of Stirling under equivalent conditions since March 2023 and are regularly genotyped to check their genetic identity has been maintained. The wasps are given black bean aphid (*Aphis fabae*) colonies to parasitize; they are able to parasitize all instars of aphid but prefer first instar nymphs [19]. Female wasps lay a single egg inside each aphid (they are solitary endoparasitoids) and the host continues to grow as the wasp larva develops (they are koinobionts [19]). In the laboratory, aphids are maintained on broad bean seedlings (*Vicia faba*). All insects are kept in climate-controlled chambers at 20°C, 70% relative humidity on a 16 : 8 h light : dark cycle. Under these conditions, the generation time of *L. fabarum* is 12−14 days.

**Table 1.** Number of first-generation G0 females that remained virgin (V), accepted a mating (M: successful matings), did not accept a mating because the male did not attempt courtship, or because the female rejected the male. (Also shown are the proportion of male–female introductions that resulted in a successful mating by line. A significantly lower proportion of pairings involving asexual females resulted in successful mating (*$x_{1}^{2}$*= 10.430, *p* = 0.0012).)

line	collection year	collection location	remained virgin	attempted matings	successful matings	no attempt	rejected	proportion attempts successful
CHC17-66	2017	Thurgau (CH)	5	5	5	0	0	1
CV17-84	2017	Zurich (CH)	4	6	5	0	1	0.83
IL06-658	2006	Cambridge (GB)	3	5	5	0	0	1
IL07-64	2007	Zurich (CH)	11	25	10	4	11	0.4
IL09-348	2009	Genève (CH)	10	14	8	1	5	0.57
IL09-402	2009	Lagenthal (CH)	10	20	10	4	6	0.5
IL09-554	2009	Aesch (CH)	5	5	5	0	0	1
sexual	2012	Various Switzerland (CH)	18	23	22	1	0	0.96

### 2.3. Experimental design

**Figure 1.**
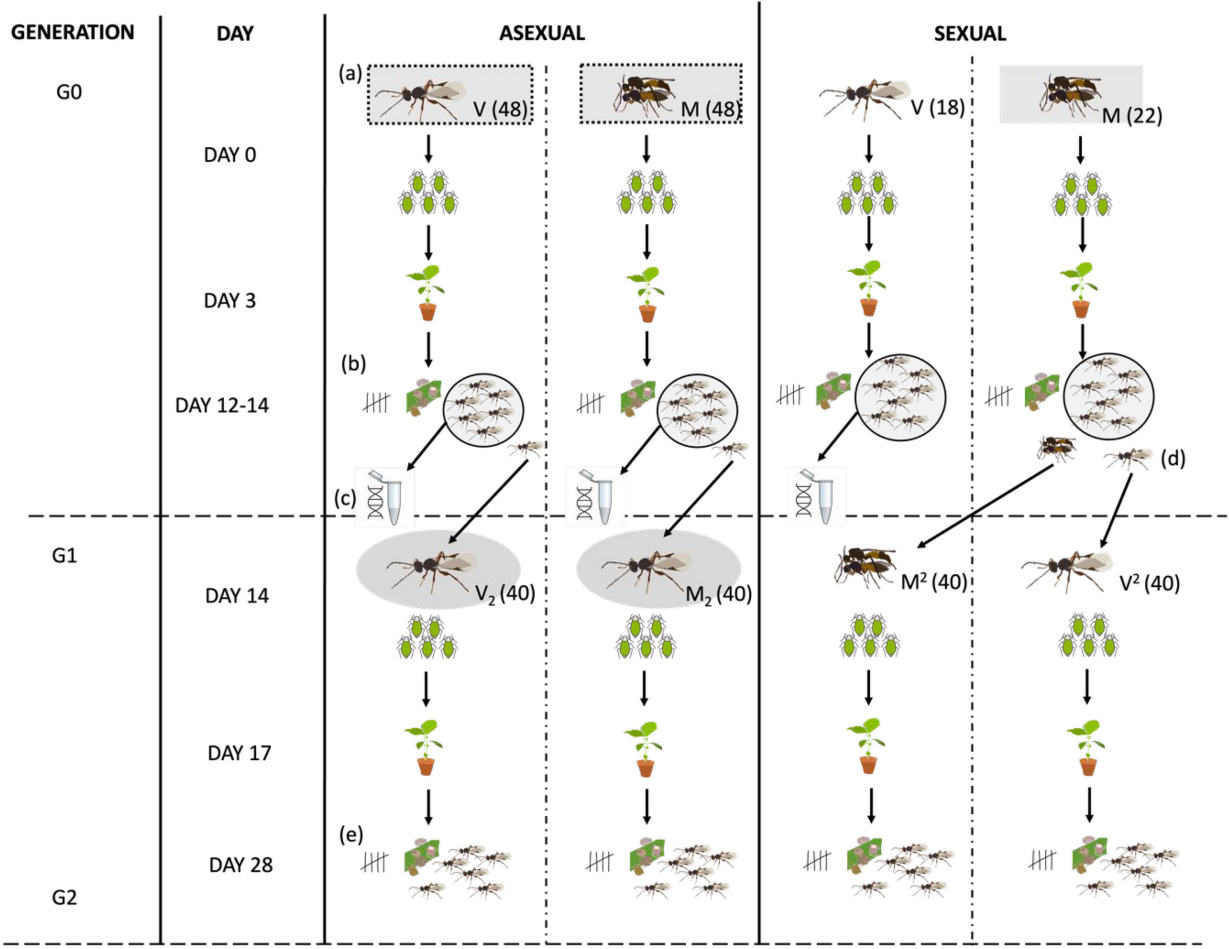
Schematic of the experimental design showing (a) the two treatments (V and M; sample sizes per treatment shown in brackets after treatment names) that sexual and asexual females were subjected to initially (G0) and how offspring were either (b) sexed and counted and preserved in ethanol (c) for DNA extraction or used to set up G1 (d; two daughters were taken from each sexual G0 mother). When the second generation of wasps emerged (e) they were sexed and counted. Boxes around focal wasps (a,c) indicate treatments which were compared to each other to estimate the costs of male production for obligate sexuals compared to asexuals (grey rectangles) and facultative sex versus asex (grey ovals).

To measure the benefits and costs of sex in *L. fabarum,* I assayed offspring production by obligately sexual and asexual *L. fabarum* females (referred to as G0) which were exposed to a male (from the obligately sexual population), M, or remained virgin, V (figure 1a). All females (including asexuals) in the M treatment readily copulated with the male, so I refer to these females as ‘mated’. To test for longer-term costs of mating (and potentially facultative sex), I repeated the same fitness assays with the unmated daughters of V and M asexual females (figure 1c; V_2_ and M_2;_ referred to as G1 females). The fitness measures I used were: (i) the number of aphid mummies (parasitized dead aphids) produced; and (ii) the number of mummies from which adult wasps successfully emerged (figure 1b,d). Emerging wasps (figure 1b,d) were also sexed in order to test whether asexual females avoid the ‘twofold’ cost of male production regardless of their mated status. To test whether asexual females that mated reproduced by facultative sex (i.e. used sperm to fertilize their eggs), I genotyped full broods of emerged wasps (figure 1b,d) to test the presence of sexual alleles in their offspring.

To assess the cost of male production and determine whether it applies to facultative (mated asexual) and obligately sexual females I looked at the number of sons and daughters produced by V, M and V_2_ and M_2_ females. To estimate the cost of sex in the case of obligate sex I compared son and daughter production between G0 obligately sexual mated females and all (V and M) G0 asexual females (figure 1, grey boxes). To test whether the cost of male production also applies to facultatively sexual females (asexual M females) I compared son and daughter production of (i) G0 asexual V and M females (figure 1, dashed outlined boxes), and (ii) G1 asexual V_2_ and M_2_ females (figure 1, grey ovals). I predicted that the only obligately sexual females (sexual M) would pay the cost of male production, producing approximately 50% male offspring and as a consequence would produce around half as many daughters as asexual females (asexual M and V). I predicted male production would be negligible for all asexual females, including those which mated with a sexual male (M) or whose mother had mated (M_2_). In §2.6, I outline the statistical models applied to make these comparisons.

### 2.4. Experimental set-up

#### 2.4.1. Generation 1: standardized rearing

I set-up a ‘G-1’ generation to standardize rearing conditions for females to be used in experiments; recently emerged females were removed from stock for all lines (seven asexual and one sexual) and 20 females per line were transferred onto broad bean seedlings infested with *A. fabae* in cellulose bags (Celloclair, Liestal, Switzerland). To generate enough males for the mating treatments, 20 virgin sexual *Lysiphlebus* females were allowed to parasitize an aphid colony. Virgin sexual females laid only unfertilized eggs that develop as haploid males. To ensure virginity, parasitized aphid ‘mummies’ were removed from infested plants in the sexual stock cage before the wasps emerged and were isolated in cellulose pill capsules. When the wasps emerged, 20 females were transferred onto broad bean seedlings infested with *A. fabae* in a cellulose bag. This process was repeated for each asexual line and the sexual population to generate focal females, but emerged females were removed from stock, and not isolated as mummies. This process was repeated with freshly emerged wasps every day for 3 days to stagger the emergence of focal wasps to increase the sample size by conducting the experiment across three blocks.

#### 2.4.2. Generation 0: mating treatments

Ten to 12 days after aphids were exposed to the G-1 wasps I checked the seedlings for the presence of parasitized aphid ‘mummies’ and isolated any that I found into cellulose pill capsules. These were labelled with the line ID, whether the mother was mated or virgin (for sexuals) and the date the mummy was isolated. Mummies were checked regularly for emergence. On the peak emergence days (day 13) I selected focal females and males and assigned them to mating treatments. I set up between 3 and 11 females per treatment for each asexual line and 18 and 22 sexual females for the virgin and mated treatments respectively resulting in a total sample size of *n* = 126.

Asexual female wasps from seven lines and sexual females were assigned to either mated (M) or virgin (V) treatments. For the M treatments, a male produced by a sexual virgin female was introduced into the pill capsule with the female, the pair was observed continuously for 30 minutes or until copulation occurred. In *L. fabarum* it is possible to determine successful mating visually, the female becomes quiescent and lowers her antenna and the male is able to insert his aedeagus into her vagina. Females often reject male mating attempts in this species by curling their abdomen under and moving rapidly. For all M females, I recorded whether males attempted copulation and if so whether females rejected or accepted the mating attempt. For M females that accepted copulation, the male was removed from the pill capsule and the female was retained (I did not assay the fitness of M females which did not copulate in this experiment). For the V treatments, no male was added to the females pill capsule, to control for potential mechanical costs of mating, V females were gently moved around in their pill capsule using a paintbrush.

On each day that wasps were put into M and V treatments (over 3 days) once all females had been subjected to their mating treatment, they were transferred onto broad bean leaves cut into discs and embedded in 6 ml 1% agarose gel in an insect rearing dish with a mesh ventilation hole (diameter, 15 mm depth; SPL Life Sciences, Pocheon-si, South Korea). Leaf discs had been infested with five adult aphids 5 days previously. On the day of the experiments, the adult aphids were removed from the leaf disc and the nymphs that they produced were counted and numbers recorded (mean 62 ± 25 nymphs per disc). Wasps on leaf discs were checked every day at 9.00−10.00, 12.00−13.00 and 17.00−18.00 for 3 days after they were set up and any dead females were recovered, and their time and date of death recorded. After 3 days, the leaf disc was removed from the agar and transferred onto a two-week-old broad bean seedling in a cellophane bag. If the female wasp was still alive, she was transferred onto the seedling along with the aphids.

Twelve days after the aphids had been exposed to the wasps, I cut the stem of the seedling and closed the bag (still containing the seedling) with a bulldog clip. Cutting the plant resulted in any unparasitized aphids dropping off the plant before wasps started to emerge. The bags were checked each morning from days 12 to 16 and any emerging wasps were collected. I collected one female from each replicate (with the exception of sexual virgin females, which do not produce daughters) to start generation G1 (see below). The rest of the G1 brood were collected into 99% ethanol for genotyping. Bags containing mummies were retained and the mummies counted after all wasps had emerged.

#### 2.4.3. Generation 1: daughters of mated and virgin females

To test whether mothers mated status influences the fitness of asexuals, I assayed the fitness of the daughters produced by G0 asexual females in the M and V treatments and G0 sexual females in the M treatment (sexual V females produced only sons). Generation G1 asexual females which had emerged into cellulose bags were collected and subjected to the same protocol as G0 females, but G1 focal females all remained virgin; they were the daughters of asexual females that had either mated (labelled M_2_) or remained virgin (labelled V_2_). I took two G1 daughters from each mated sexual G0 female, one was isolated before emergence and so remained virgin (V_2_), and the other was allowed to emerge and mate naturally in the cellulose bag (M_2_: figure 1). Females were placed onto leaf discs for 3 days, which were then moved onto seedlings. The number of aphid nymphs and wasp survival on leaf discs was also recorded for these G1 females. I counted the number of mummies and emerged wasps produced by these G1 females. For each combination of line by treatment set-up between 3 and 11 focal females, resulting in a total sample size of *n* = 80 G1 females.

### 2.5. Assaying sperm use by asexual females

To test whether asexual females used sperm from sexual males after they mated I genotyped 41 whole broods of offspring (eight broods from asexual virgin females, 29 broods from mated asexual females and four broods from sexual females) using a single microsatellite marker (*Lysi07* [18]) which exhibits complete linkage with the asexuality-inducing allele [16]. DNA was extracted in a pooled reaction (up to 10 wasps from one brood per reaction) using the Qiagen DNAeasy blood & tissue kit (QIAGEN Inc, Hilden, Germany). After extraction, DNA was amplified in a multiplex polymerase chain reaction (PCR) using fluorescently labelled *Lysi07* primers. PCRs were carried out using a Qiagen Multiplex PCR kit (QIAGEN Inc., Hilden, Germany) in 11 μl reactions and contained: 1 μl DNA template, 10 μl PCR mastermix (made up 1× QIAGEN Multiplex PCR mix and 0.1 µM of each locus-specific labelled (forward) and unlabelled (reverse) primer). PCR conditions were as follows: initial denaturation at 95°C for 15 min, followed by 30 cycles of 94°C for 30 s, 58°C for 90 s, 72°C for 60 s and final extension at 60°C for 30 min. PCR amplification was performed using a DNA Engine Tetrad® Thermal Cycler (MJ Research, Bio-Rad, Hemel Hempstead, Herts, UK). PCR products were then sent to MRC PPU(MRC Protein Phosphorylation and Ubiquitylation Unit) DNA sequencing and services at the University of Dundee for fragment analysis. This involves capillary electrophoresis which provides a series of different coloured peaks which vary in location on an *x*-axis based on the size of the microsatellite fragment. I scored these peaks using the software Geneious™. For *Lysi07,* peaks scored at 184 are found exclusively in asexual wasps and 192 in sexual wasps. When a sample had scores at both 184 and 192, it indicated the presence of both sexual and asexual alleles, suggesting that an asexual wasp had mated and used sperm.

### 2.6. Statistical analysis

#### 2.6.1. Parasitism failure, mummy production and wasp emergence

To test whether mated status (and reproductive mode) were related to reproductive success I ran three sets of generalized linear mixed models (GLMMs) with the following response variables: (i) parasitism failure (binomial, scored as 1 if no mummies were recovered; using the glmer function in the lme4 package in R [20]); (ii) number of mummies (negative binomial, count of the number of mummies recovered from each replicate; using *glmmTMB* in R [21]); and (iii) proportion of mummies that emerged as adult wasps (binomial, modelled using the cbind function in R and using *glmmTMB*).

The G0 models included reproductive mode (sexual or asexual), mated status (virgin or mated) and their interaction effect as fixed factors. Covariates were also fitted; these were the number of aphid nymphs on the leaf disc and female wasp survival (in hours; up to a maximum of 72 h for all wasps which survived until transfer to seedling). I also included a random effect of line ID nested within reproductive mode. Block was not included in any model; treatments were replicated evenly across daily blocks and in no case did I find a significant effect of block on any outcome variable, nor did inclusion of block in any models influence the results. Broods that were recorded from two G0 virgin sexual females were removed from the analyses as female offspring were recovered (virgin sexual females only produce unfertilized eggs which develop as males) suggesting mislabelling.

The G1 models were similar but the data were only from asexual females, so no fixed effect of reproductive mode was fitted. The only fixed effects were mother’s mated status (mated or virgin), survival of the focal female and number of nymphs (as covariates) and line ID was included as a random effect. The package *DHARMa* [22] was used to check that all models were appropriately specified.

#### 2.6.2. Daughter production and the cost of males

To compare the traditional ‘twofold’ cost of sex (male production), I tested whether obligately sexual females produced fewer daughters than all asexual females (grey boxes in figure 1). To do this I ran a model using data from G0 females, with the number of female offspring produced as the outcome variable (negative binomial in *glmmTMB*). Data from virgin sexual females were not used for this model (as virgin sexual females produce only male offspring), but data from M sexual and asexual and V asexual G0 females were included. This G0 model included a fixed effect of reproductive mode. The number of male offspring produced was also included as a covariate, and as an interaction with reproductive mode, to test for a trade-off between son and daughter production.

I ran two additional models to test whether facultative sex (by normally asexual females) imposes a cost owing to male production. These models had the same structure but included data either from only G0 or only G1 asexual females and included fixed effect of mated status (or mother’s mated status) and the number of male offspring produced (as a covariate), as well as their interaction effect. The G0 model tested whether asexual females that mated produced fewer daughters than females which remained virgin (grey boxes with dashed outlines in figure 1). The G1 model tested whether females with mated mothers produced the same number of daughters as females with virgin mothers (grey ovals in figure 1). As with the other models mothers’ survival and number of nymphs were included as covariates and random effects were line ID nested within reproductive mode or and line ID only (if only data from asexual females was used).

## 3. Results

In the first generation (G0), 103 females were offered a male to mate with (M) and 66 were not (they remained virgin, V). Of the 103 females assigned to the M treatment, 70 mated. Thirty-three M females did not mate either because males failed to initiate courtship (*n* = 10), or the female did not signal receptivity (*n* = 23). The 70 M and 66 V female wasps were given aphids to parasitize. Table 1 shows the number of females which remained virgin, mated or did not mate (owing to rejection or no attempt) by line.

Of the 136 G0 females that were given aphids to parasitize, 58 died before they were transferred onto the seedlings and 70 survived. In the second generation (G1), I took the daughters of 40 M and 40 V G0 asexual mothers and put them on leaf discs. Of the 80 G1 females put onto leaf discs 57 survived and 23 died before being transferred to seedlings 72 h later (note that there was no effect of mating treatment on wasp survival in the first 72 h in either the G0 or G1 generations: G0 (effects of reproductive mode and mated status): log-rank *$\chi_{3}^{2}$*= 1.4, *p* = 0.70 and G1 (mated status only): log-rank $\chi_{1}^{2}$= 2.8, *p* = 0.33).

### 3.1. Sperm use

Forty-one broods were genotyped in total (see the electronic supplementary material, table S1 for a summary of brood genotypes broken down by treatment and line). All broods produced by sexual females were homozygous for the sexual allele (peak 192) at *Lysi07*. Broods produced by asexual females were significantly more likely to test positive for sexual alleles if the female had mated (M) than if she was virgin ($\chi_{1}^{2}$= 4.56, *p* = 0.03); 9 out of 30 broods produced by mated asexual females were homozygous for the asexual allele (peak 184), 19 out of 30 were heterozygous for sexual and asexual alleles (peaks 184/192) and two were homozygous for the sexual allele (192; most likely owing to a PCR failure/scoring error or misnumbering of a replicate as there is no evidence for introgression of sexual alleles in the stock lines these females were derived from). Only one brood produced by a virgin asexual female was scored as positive (heterozygous) for the sexual allele (suggesting either mislabelling or contamination between samples); the other seven V asexual broods were homozygous for the asexual allele.

### 3.2. Parasitism success

**Figure 2.**
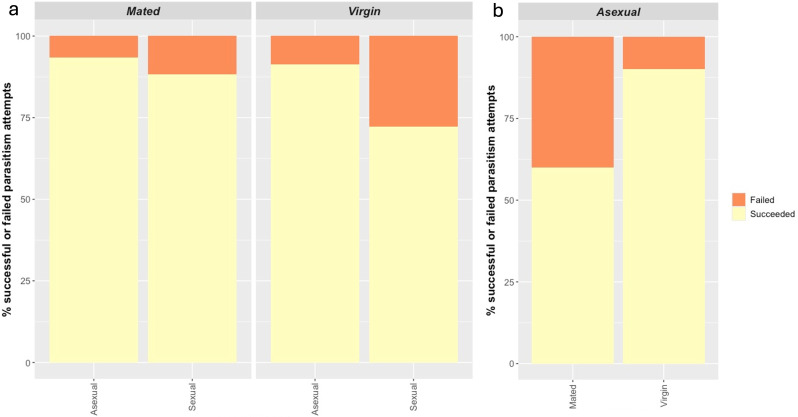
Bar plot showing the effect of mated status on per cent parasitism success in (a) G0 across seven asexual lines and one outbred sexual line of *L. fabarum*. In G0, sexual females were more likely to fail to parasitize than asexuals but there was no effect of mated status and (b) G1 asexual daughters of G0 females; daughters of mated females were significantly more likely to fail to parasitize than daughters of virgin females.

In some cases, no mummies were recovered from aphids that were exposed to *L. fabarum* females, suggesting that these females either failed to oviposit, or their eggs failed to hatch/larvae to develop resulting in complete reproductive failure. In G0, 100 out of 126 females successfully parasitized aphids and produced adult offspring. A few individuals successfully produced a small number of mummies but no adult offspring (12), these females were excluded from the analyses below as I did not dissect the uneclosed mummies to determine whether the larvae died before emergence or had entered diapause. In the first generation G0, sexual females were more likely to fail to parasitize aphids than asexual females ($\chi_{1,124}^{2}$ = 4.49, *p* = 0.03). Mating had no effect on G0 females ability to parasitize and this was true for both sexual and asexual females ($\chi_{1,124}^{2}$ = 1.54, *p* = 0.21*.* Interaction effect between reproductive mode and mated status: $\chi_{1,124}^{2}$ = 0.42, *p =* 0.52; figure 2a).

Fifty-four out of 80 G1 daughters of asexual G0 females successfully parasitized aphids and produced adult offspring. Of these, six females successfully produced a small number of mummies but no adult offspring (again, females which produced mummies but not adult offspring were removed from the analysis below). In G1, there was a large effect of mothers mated status on parasitism success. The G1 daughters of mated G0 asexual females were 25% more likely to fail to parasitize aphids than the daughters of virgin V females ($\chi_{1,79}^{2}$ = 7.43, *p* = 0.0.006*;* figure 2b).

### 3.3. Mummy production and emergence

**Figure 3.**
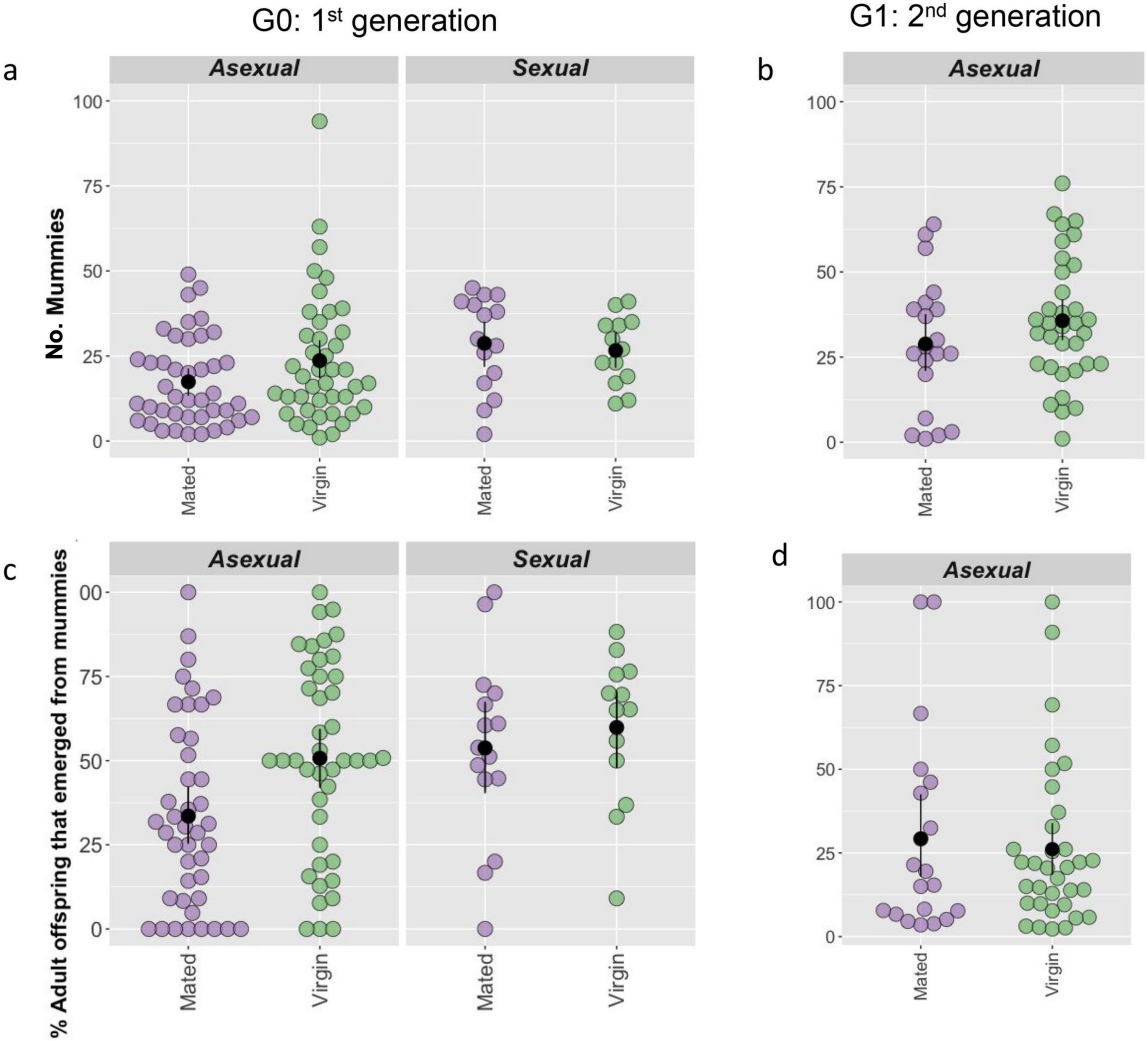
Number of mummies produced (a,b) and per cent of mummies adult wasps emerged from (c,d), in G0 (a,c) and G1 (b,d). G0 sexual females produced more mummies than asexual females (a) which were more likely to emerge as adult wasps (c). Mummies produced by virgin G1 mothers (sexual and asexual) were also more likely to emerge as adult wasps (c). For asexual G1 females, there was no effect of mothers mated status on mummy production (b) or the proportion of mummies that successfully emerged (d). Black points are means and error bars represent standard errors estimated by non-parametric bootstrapping.

Although sexual females in G0 were more likely to fail to parasitize any aphids, when I considered only the females that did parasitize, sexual females were at an advantage as they produced more mummies ($\chi_{1,98}^{2}$ = 5.04, *p* = 0.03; figure 3a) more of which produced a wasp ($\chi_{1,98}^{2}$ *=* 4.41, *p* = 0.03; figure 3c) than their asexual conspecifics. While sex had an effect on mummy production in G0 there was no effect of mating; virgin and mated females produced the same number of mummies ($\chi_{1,98}^{2}$ = 0.30, *p* = 0.59), regardless of whether they were sexual or asexual (interaction: $\chi_{1,98}^{2}$ = 0.29, *p* = 0.59*;* figure 3a). There was an effect of mating on adult offspring production though; virgin G0 mothers were more successful ($\chi_{1,98}^{2}$ *=* 3.85, *p* = 0.05*;* figure 3c) for sexual and asexual mothers (interaction: $\chi_{1,98}^{2}$ *=* 0.78, *p* = 0.38).

These results were not replicated in the second generation, there was no effect of G0 mother’s mated status on the number of mummies their G1 daughters produced ($\chi_{1,52}^{2}$ = 2.41, *p* = 0.121; figure 3b) or the proportion of these mummies from which a wasp emerged ($\chi_{1,52}^{2}$ = 0.21, *p* = 0.65; figure 3d).

#### 3.3.1. Daughter production and the cost of producing males

To test whether the classic ‘twofold’ cost of male production applies in *L. fabarum* I looked at daughter production by focal females. In G0, obligately sexual females did not produce fewer daughters than asexual females (M or V; $\chi_{1,98}^{2}$ *=* 0.02, *p* = 0.90)*.* Contrary to expectations females that produced more males did not produce fewer daughters (number of sons: $\chi_{1,98}^{2}$ *=* 0.68, *p* = 0.40)*,* a pattern that was the same for sexual and asexual females (reproductive mode*number male offspring: $\chi_{1.98}^{2}$ *=* 3.23, *p* = 0.07)*.* I ran a second model with sexual females removed to test whether facultative sex by G0 mated asexual females imposes a cost in terms of male production, but again M and V females produced the same number of daughters ($\chi_{1,71}^{2}$ = 0.49, *p* = 0.48). Son production was very low for M and V asexual females in G0 (<5 per mother) and there was no evidence that females which produced more males produced fewer daughters (mated status: $\chi_{1,71}^{2}$ *=* 0.49, *p* = 0.48; male production; *χ*^2^_1,71_*=* 3.22, *p* = 0.07; mated status*number male offspring $\chi_{1,71}^{2}$ = 0.36, *p* = 0.55).

I found the same pattern in the second generation (G1); male production was again very low for all asexual females and had no effect on daughter production ($\chi_{1,52}^{2}$ *=* 1.88, *p* = 0.17) regardless of mothers mated status (mothers mated status*number male offspring: $\chi_{1,52}^{2}$ *=* 0.65, *p* = 0.42). Importantly, the G1 daughters of mated G0 females (some of which are facultatively sexual) produced the same number of daughters as females produced asexually by virgin G0 mothers ($\chi_{1,52}^{2}$ = 2.24, *p* = 0.14). Taken together, these results from G0 and G1 suggest that ‘all-else-is-not-equal’ in this system, so the twofold cost of male production does not constrain the fitness of (obligately or facultatively) sexual females compared to asexuals in *L. fabarum*.

## 4. Discussion

Populations of the aphid parasitoid *L. fabarum* vary in their reproductive mode: asexual (parthenogenetic) reproduction dominates across Europe, but sexual populations persist in sympatry with asexuals in some regions [14,15]. In this study, I show for the first time, to my knowledge, that asexual *L. fabarum* females are sexually functional and can use sperm to fertilize eggs and produce daughters. I found that some females from all seven asexual *L. fabarum* lines assayed were facultatively sexual; they mated with sexual males and produced offspring with genetic contributions from both parents. However, asexual females that reproduced sexually did not get the best of both worlds, in terms of short-term benefits of parthenogenesis (higher daughter production) or genetic benefits of sex (increased fecundity or offspring emergence). In fact, the pattern was the opposite, facultatively sexual females suffered high costs of reproductive failure associated with sex *and* lower fecundity associated with asexual reproduction; they get the worst of both worlds.

A main short-term benefit of asexual reproduction is that it eliminates the ‘twofold cost of males’. In *L. fabarum,* asexuals do occasionally produce male offspring, Sandrock & Vorburger [16] found that around 1 in every 3000 asexual wasps is male in the laboratory. In the current study, I also found low rates of male production by *all* asexual females, but crucially asexual females did not produce more daughters than sexual females. This is because the assumption that 'all-else-is-equal’ does not hold; obligately sexual females had higher fecundity than females from asexual lines, such that despite producing many more sons they produced the same number of daughters as asexuals, suggesting a long-term genetic benefit of sex. However, I also found that the costs of sexual reproduction in this system extend beyond male production. The elevated rates of reproductive failure in the daughters of facultatively sexual females (approx. 40%) suggest additional costs, possibly related to genetic incompatibilities or outbreeding depression. For instance, some of the daughters of facultatively sexual females may be triploid with low fertility [15] or outbreeding depression may limit the fitness of hybrid offspring. ‘Genetic slippage’ is a form of outbreeding depression which is particularly relevant under rare facultative sex. It occurs when co-evolved combinations of genes that normally work well together are broken up by sex, creating new genotypes which have low fitness [3–8,12]. Sex and recombination can produce novel multilocus genotypes that perform better than the parental genotypes in some environments, but genetic slippage can mean that they perform much worse [12]. In this study, it may be that the new genotypes created by sex were costly for *L. fabarum,* and this cost may have been more severe under facultative sex because of the greater genetic distance between asexual females and sexual males.

An additional cost that is important to consider in the case of facultative sex in *L. fabarum* is that of genome dilution. The cost of male production, rather than genome dilution, is widely acknowledged to be the main cost of obligate sex [4], but genome dilution becomes relevant when genes that determine the mode of reproduction are potentially diluted. Genome dilution only applies when genes for sexual and asexual reproduction come together, which is precluded when asexual and sexual reproduction are obligate. Under facultative sex genome dilution can occur if some offspring produced through facultative sex inherit different copies of the allele which determines reproductive mode [17]. As such, while the ‘twofold cost of males’ does not appear relevant for *L. fabarum,* a comprehensive comparison of fitness requires more extensive individual-level genotyping of whole broods to determine the proportion of female offspring that are produced parthenogenetically versus sexually when normally asexual females mate. Such studies would also allow for more precise measurements of the other costs of sex; genotyping individual mothers and offspring (and measuring genetic distance between the parents) will provide estimates of the extent to which triploidy and outbreeding contribute to the costs of facultative sex. Similarly, repeating this study but using rare males that are produced by asexual females will allow for more explicit tests of the genetic slippage hypothesis, as facultative sex between more closely related mates should in theory be less costly.

Asexual reproduction is thought to have first evolved approximately 0.5 million years ago in *L. fabarum,* arising through the inheritance of a single recessive allele which results in the haploid products of meiosis I fusing to form diploid gametes which develop without fertilization (central-fusion automixis [17]). Although asexuality may have arisen 0.5 million years ago, it is unlikely that the asexual lineages I tested have existed for so long [17]. The asexual lines used in this experiment were all collected in areas of sympatry (notably 6 out of 7 asexual lines were collected in the same region that the outbred sexual population was collected from), but they have been reared in the laboratory for 7−18 years without sex. Despite the lack of sex (potentially for up to 400 generations) some females from all seven asexual *L. fabarum* populations exhibited sexual behaviour and were able to integrate paternal DNA into the female offspring they produced. Moreover, the propensity to engage in sex and mating (and the costs that accrue as a result of facultative sex) do not appear to be related to how long the lines had been maintained in the laboratory for, or where they are collected from.

The maintenance of sexual traits in *L. fabarum* makes them unique compared to many other asexual eukaryotes, where sexual traits such as mate location, attraction, allowing copulation and storing sperm decay rapidly owing to high costs of maintenance [23]. In other parasitoid species that have reverted to asexual reproduction owing to parthenogenesis inducing *Wolbachia* infection, females no longer produce costly volatile chemoattractants which render them attractive to males [24–26]. What remains unclear is why sexual traits have persisted in asexual *L. fabarum* despite being costly in terms of reproductive success. One possibility is that facultative sex is maintained as a bet-hedging strategy, safeguarding against unpredictable environmental conditions. By producing genetically variable offspring, some may be better suited to fluctuating conditions, even if many suffer from reduced, or even zero, fitness [27–30]. Under bet-hedging, sex is a risk-averse strategy associated with moderate fitness under a range of environments, while asexual reproduction could provide big benefits under a stable environment but risks extinction if conditions change. The life histories of aphid parasitoids like *Lysiphlebus* might increase the importance of facultative sex as a bet-hedging strategy because offspring produced at the end of summer must survive overwinter as diapause pre-pupae and reproduce the following spring, in drastically different environmental conditions from their parents. Facultative sex might increase survival and reproduction of overwintering individuals by creating novel genotypes which thrive in novel conditions [27–30].

Mating systems and population dynamics characteristic of parasitoid species like *L. fabarum* may act alongside bet-hedging to increase the likelihood of, and reduce the costs associated with, facultative sex. Aphid parasitoids experience increased abundance over the season as hosts become more numerous, until populations crash later in the summer [30]. At the end of the season, the abundance of *L. fabarum* females in parasitized aphid colonies can become very high, which could have a number of effects on the rate of facultative sex and its consequences. First, high population densities are expected to increase the likelihood that normally asexual females will encounter and mate with rare asexual males (or sexual males in regions of sympatry). Second, mating between kin is common in parasitoids because many females parasitize aphids in the same colony that they emerged from (they are quasigregarious), which may counter the detrimental effects of genetic slippage (i.e. they are less likely to produce low fitness recombinants with closely related mating partners: [31]). Genetic slippage is essentially a form of severe outbreeding depression [32], and so its costs may be reduced under inbreeding. Third, the main cost of sex (increased reproductive failure) may be less important at certain times in the season. For instance, when aphid populations crash towards the end of the season [33], the costs of host finding and host competition may constrain parasitoid fitness to a greater extent than the costs of sex.

When viewed in the light of the natural history of aphid parasitoids like *L. fabarum,* the results of this study suggest that relatively frequent sex, potentially between closely related males and parthenogenetic females, may be responsible for maintaining high levels of genetic variation and heterozygosity in asexual *L. fabarum.* If sex occurs mainly late in the season, it is also likely to be fairly synchronous, mirroring the reproductive phenology of their cyclically parthenogenetic aphid hosts. When normally parthenogenetic individuals all reproduce sexually at the same time in this way, Kokko [12] has shown that facultative sex is more stable and robust to the invasion of obligate asexual reproduction. Synchronous seasonal sex between siblings may explain not only how heterozygosity and allelic diversity are maintained in asexual *L. fabarum* [14,15] but also why sex and its associated traits have persisted. Future work should measure the frequency of sex in the field and its associated costs, including genome dilution and inbreeding, to reveal the full extent of facultative parthenogenesis and its evolutionary significance in *L. fabarum*.

## Data Availability

All data, code and supplementary information can be accessed here [34]. Supplementary material is available online [35].
